# Pharmacoeconomics of PCSK9 inhibitors in 103 hypercholesterolemic patients referred for diagnosis and treatment to a cholesterol treatment center

**DOI:** 10.1186/s12944-016-0302-8

**Published:** 2016-08-18

**Authors:** Parth Shah, Charles J. Glueck, Vybhav Jetty, Naila Goldenberg, Matan Rothschild, Rashid Riaz, Gregory Duhon, Ping Wang

**Affiliations:** From the Cholesterol, Metabolism, and Thrombosis Center, Jewish Hospital of Cincinnati, Cincinnati, USA

**Keywords:** PCSK9, Evolocumab, Alirocumab, Cholesterol, Lipids, Pharmacoeconomics, Heterozygous familial hypercholesterolemia (HeFH), Cardiovascular disease (CVD)

## Abstract

**Background:**

PCSK9 inhibitor therapy has been approved by the FDA as an adjunct to diet-maximal tolerated cholesterol lowering drug therapy for adults with heterozygous familial hypercholesterolemia (HeFH) or clinical atherosclerotic cardiovascular disease (ASCVD) with suboptimal LDL cholesterol (LDLC) lowering despite maximal diet-drug therapy. With an estimated ~24million of US hypercholesterolemic patients potentially eligible for PCSK9 inhibitors, costing ~ $14,300/patient/year, it is important to assess health-care savings arising from PCSK9 inhibitors vs ASCVD cost.

**Methods:**

In 103 patients with HeFH, and/or ASCVD and/or suboptimal LDLC lowering despite maximally tolerated diet-drug therapy, we assessed pharmacoeconomics of PCSK9 inhibitor therapy with lowering of LDLC. For HeFH diagnosis, we applied Simon Broome’s or WHO Dutch Lipid Criteria (score >8). Estimates of direct and indirect costs for ASCVD events were calculated using American Heart Association (AHA), U.S. DHHS, Healthcare Bluebook, and BMC Health Services Research databases. We used the ACC/AHA 10-year ASCVD risk calculator to estimate 10-year ASCVD risk and estimated corresponding direct and indirect costs. Assuming a 50 % reduction in ASCVD events on PCSK9 inhibitors, we calculated direct and indirect health-care savings.

**Results:**

We started 103 patients (58 [56 %] women and 45 [44 %] men), on either alirocumab (62 %) or evolocumab (38 %), median age 63, BMI 29.0, and LDLC 149 mg/dl. Of the 103 patients, 28 had both HeFH and ASCVD, 33 with only ASCVD, 33 with only HeFH, and 9 had neither. Of the 103 patients, 61 had a first ASCVD event at median age 55 and on best tolerated cholesterol-lowering therapy median LDLC was 137 mg/dl. In these 61 patients, total direct costs attributable to ASCVD were $8,904,361 ($4,328,623 direct, $4,575,738 indirect), the median 10-year risk of a new CVD event was calculated to be 13.1 % with total cost $1,654,758. Assuming a 50 % reduction in ASCVD events on PCSK9 inhibitors in our 61 patients, $4,452,180 would have been saved in the past; and future 10-year savings would be $1,123,345.

**Conclusion:**

In the 61 CVD patients, net costs/patient/year were estimated to be $7,000 in the past, with future 10-year intervention net costs/patient/year being $12,459, both below the $50,000/year quality adjusted life-year gained by PCSK9 inhibitor therapy.

## Background

Many patients with elevated LDLC fail to achieve treatment targets [1–3], because of statin intolerance [4, 5], expense, lack of insurance coverage, or variations in statin availability across states in insurance, race, and ethnicity [1]. With LDLC lowering potency well beyond statins, PCSK9 inhibitors now offer the promise of optimizing LDLC in a majority of patients with heterozygous familial hypercholesterolemia (HeFH), cardiovascular disease (CVD), and statin intolerance [6–11]. The PCSK9 inhibitor class of medications allows patients to attain LDLC levels that were previously unattainable with maximal diet-drug regimens [6, 10–13]. Preliminary controlled clinical trials, though not powered to assess cardiovascular outcomes, showed approximately a 50 % risk reduction in cardiovascular events [14, 15].

Whether and to what degree health care insurers will facilitate approval of PCSK9 class of medications [11, 14, 16] at an annual price of $14,000–14,600 per patient may ultimately be determined by the outcomes of placebo-controlled trials of hard CVD endpoints and all-cause mortality [13] or surrogate CVD endpoints such as regression or non-progression of atherosclerosis by intravascular ultrasound. Overall costs to society also need to include analysis of any potential adverse effects arising from PCSK9 inhibitor use.

Of 734 patients referred to our Cholesterol Center for diagnosis and treatment of high LDLC and/or CVD, with LDLC ≥70 mg/dl despite maximally tolerated cholesterol lowering therapy, we recently reported [17] that 30 % were eligible by FDA [18] and insurance carrier criteria for PCSK9 inhibitor therapy [11, 14, 18]. In the general population of the US [19], the CDC recently reported that 36.7 % (78 million) adults (>21 years) were eligible for cholesterol-lowering medication, but only 55 % were taking a cholesterol lowering medication of whom ~90 % were taking a statin. If 30 % of the 78 million hypercholesterolemic adults in the general US population [19] were, as in our study of hypercholesterolemic subjects [17], eligible by FDA [18] and insurance carrier criteria for PCSK9 therapy, this would include ~11 % of the adult population or 23.4 million adults.

Given current pricing of $14,000–14,600 per patient per year, annual United States PCSK9 inhibitor costs might approximate $185–$342 billion, reflecting the use of an expensive specialty drug for endemic CVD, the leading cause of mortality in the USA [20, 21]. In 2011, annual costs for CVD and stroke were estimated to be $320.1 billion [22]. If, speculatively, CVD and stroke incidence could be halved by PCSK9 therapy [11, 14, 16], direct annual savings would be estimated to be $160 billion, and indirect annual savings might be $85 billion [21], altogether $245 billion savings, in the middle of the range of estimated PCSK9 inhibitor costs of $185–342 billion [17]. Programs targeted to prevention of CVD should provide substantial overall cost savings [23, 24].

Answers are needed for major questions regarding PCSK9 inhibitor therapy including whether the PCSK9 inhibitors will significantly reduce morbid and mortal CVD events in hypercholesterolemic patients beyond the best currently available diet-statin therapy [25], and whether they will provide an incremental cost-effectiveness ratio [25] within a society willingness-to-pay threshold [26].

In 103 hypercholesterolemic patients, 61 with previous CVD (1st CVD median age 55, median LDLC 139 mg/dl despite maximal tolerated cholesterol-lowering therapy), we estimated direct + indirect costs of CVD, costs of estimated next 10 year CVD events, and PCSK9 inhibitor costs to assess whether PCSK9s would provide an incremental cost-effectiveness ratio [25] within a society willingness-to-pay threshold [26].

## Methods

The procedures were in accordance with the ethical standards of the responsible committee on human experimentation, approved by the Jewish Hospital Institutional Review Board. The study was carried out with signed informed consent.

Since the commercialization of PCSK9 inhibitors, starting July 2015, we have started 103 patients on either alirocumab or evolocumab. When considering PCSK9 inhibitor therapy, we had two groups of patients based on FDA and insurance criteria with suboptimal LDLC lowering. The first group of patients (*n* = 31) were those who were on “maximal-tolerated statin therapy,” and also maximum tolerated cholesterol lowering therapy (i.e., colesevelam and/or ezetimibe). The second group of patients (*n* = 72) were those who couldn’t tolerate ANY dose of two or more statins and were on maximal tolerated dose of colesevelam and/or ezetimibe. “Maximal tolerated statin therapy” includes not being able to tolerate any statin dose level.

In the 103 patients, we assessed the number approved for coverage either through commercial insurance or Medicare/Medicaid. We further characterized the approved patients based on meeting indications such as HeFH, homozygous familial hypercholesterolemia (HoFH), and/or CVD or none of the above.

In order to assess for HeFH, we applied Simon Broome’s [27] or WHO Dutch Lipid Criteria [28] (score >8) for HeFH by tendon xanthomas and LDLC >190 mg/dl and/or family history of premature cardiovascular disease and/or family history of severe hypercholesterolemia.

At the time of PCSK9 insurance coverage application, before starting PCSK9 therapy, we assessed the following patient characteristics: type and dose of PCSK9 therapy to be started, lipids and lipoprotein cholesterol levels on maximally tolerated diet and lipid lowering drugs, age, gender, BMI (body mass index), previous and current cholesterol lowering therapies, and CVD event age, if applicable. Within the CVD events group, we documented coronary artery disease, acute myocardial infarction, cerebrovascular accidents/stroke, carotid artery disease, and heart failure.

For the 61 patients who had a CVD event, the associated direct and indirect costs before starting PCSK9 therapy were calculated using U.S. Department of Health and Human Services, BMC Health Services Research, and Healthcare Bluebook databases [29–31]. For direct cost calculations, we categorized CVD patients into having coronary artery disease, acute myocardial infarction, stroke/acute cerebrovascular disease, and/or congestive heart failure and calculated average hospitalization costs as per HCUP projections [46]. In our direct cost estimations, we also included the average cost of coronary artery bypass graft, percutaneous angioplasty, carotid endartectomy, and follow-up costs for cardiac diagnostic tests (EKG, stress test, Calcium score), office visits, and cardiac rehabilitation [32]. For indirect costs calculations, we used work absenteeism and short term disability productivity losses over the years after first CVD event [29]. We also applied the present value of lifetime earnings (PVLE) model to calculate indirect costs from premature mortality within the US in our patients based on their age group [33]. We estimated savings in PVLE on PCSK9 using the PCSK9 inhibitor mortality data from Navarese et al. [34].

We used the ACC/AHA 10 year cardiovascular disease risk calculator [35] to estimate likelihood of CVD events within next 10 years, in relevance to the hypercholesterolemic population. A broad cost and benefit to society analysis was done using AHA databases [21, 22].

## Results

To date we have started 103 patients on either alirocumab (62 %) or evolocumab (38 %). Table 1 displays characteristics of this cohort of 103 patients, 58 (56 %) women and 45 (44 %) men, with median entry age 63, BMI 29.0, and mean ± SD LDLC 166 ± 55 mg/dl (median 149). Of the 103 patients, 61 had cardiac disease and/or stroke-TIAs during past 10 ± 9 years without PCSK9 therapy, Table 1. Of the 61 patients with cardio-cerebrovascular disease (CVD), 28 had both HeFH and CVD, and 33 had CVD without HeFH, Table 1. Of the 42 patients without CVD, 33 had HeFH only, and 9 had neither, Table 1.

**Table 1:** Characteristics of 103 patients started on PCSK9 inhibitor therapy

				Mean ± SD, Median At Entry					
Mean ± SD, Median	Race/ Gender	Statin intolerant	Praluent (P)/Repatha (R)	Age	BMI	TC	TG	HDLC	LDLC
All, *N* = 103	B 15 (15 %); W 88 F 58 (56 %); M 45	73 (71 %)	64 (62 %) P 39 (38 %) R	62 ± 10, 63	29.6 ± 5.4, 29	250 ± 59, 246	165 ± 86, 142	53 ± 16, 53	166 ± 55, 149
CVD, *n* = 61 1st CVD age 54 ± 11, 55	B8 (13 %); W 53 F 29 (48 %); M 32	39 (64 %)	42 (69 %) P 19 (31 %) R	65 ± 9, 66	30.1 ± 5.1, 29.7	234 ± 56, 225	168 ± 98, 139	52 ± 18, 50	150 ± 51, 139
HeFH+ CVD, *n* = 28 1st CVD age 53 ± 12, 55	B 7 (25 %); W 21 F 19 (68 %); M 9	16 (57 %)	18 (64 %) P 10 (36 %) R	59 ± 11, 61	31.5 ± 5.4, 30.9	269 ± 59, 268	159 ± 77, 133	56 ± 19, 54	181 ± 55, 191
CVD, no HeFH, *n* = 33 1st CVD age 55 ± 11, 56	B 1 (3 %); W 32 F 10 (30 %); M23	23 (70 %)	24 (73 %) P 9 (27 %) R	65 ± 10, 66	28.8 ± 4.6, 28.7	205 ± 33, 211	177 ± 113, 147	49 ± 17, 47	123 ± 26, 132
No CVD, *n* = 42	B 7 (17 %), W 35 F 29 (69 %); M 13	34 (81 %)	22 (52 %) P 20 (48 %) R	59 ± 11, 59	29.1 ± 5.9, 28.6	272 ± 56, 256	159 ± 66, 155	55 ± 14, 56	187 ± 53, 181
HeFH, no CVD, *n* = 33	B 4 (12 %), W 29 F 22 (67 %); M 11	26 (79 %)	18 (55 %) P 15 (45 %) R	56 ± 11, 57	28.8 ± 5.6, 28.5	284 ± 58, 270	165 ± 70, 156	55 ± 14, 56	198 ± 54, 189
No HeFH & no CVD, *n* = 9	B 3 (33 %); W6 F 7 (78 %); M 2	8 (89 %)	4 (44 %) P 5 (56 %) R	63 ± 13, 64	29.9 ± 7.0, 29.0	231 ± 15, 233	137 ± 48, 154	56 ± 13, 55	148 ± 17, 149

In the 103 patients, mean ± SD 10-year CVD risk calculated from the AHA/ACC calculator was 14.1 ± 12.3 %, median 11.3 % (Table 2). In the 61 patients who had sustained a cardiac disease and/or stroke-TIAs before study entry, 10-year calculated CVD risk was 15.9 ± 11.7 %, median 13.1 %. In the 42 patients who had no CVD at study entry, the next 10 year calculated CVD risk was 11.5 ± 12.8 %, median 6.8 %.

**Table 2:** Estimated^a^ 10 year risk of developing a cardiovascular disease (%)

	Mean ± SD %	Percentiles				
		10th	25th	50th	75th	90th
All 103 patients	14.1 ± 12.3	2.0	5.0	11.3	20.5	27.4
61 patients had CVD event pre study	15.9 ± 11.7	3.5	7.2	13.1	21.4	27.4
Years since 1st CVD event in the 61 patients	10.3 ± 8.9	1.4	2.9	8.2	16.3	19.5
42 patients had no CVD	11.5 ± 12.8	1.8	3.4	6.8	18.0	21.6

^a^Estimated using the ACC/AHA calculator

Follow-up lipid and lipoprotein cholesterol levels at 4 weeks on PCSK9 inhibitor therapy, along with diet were available for 94 of the 103 patients and for 56 of the 61 patients with CVD events. Median LDLC in the 94 patients fell from 152 mg/dl (on maximal tolerated cholesterol lowering therapy without PCSK9 addition) to 76 mg/dl, with the median decrement of LDLC on therapy of 79 mg/dl, percent LDLC drop from baseline 54 % (median) (Table 3). In the 56 patients with CVD disease before study entry, median LDLC fell from 141 mg/dl at entry to 60 mg/dl, with a median absolute reduction of LDLC by 79 mg/dl, median percent LDLC reduction of 57 % (Table 3). PCSK9 therapy led to a decrement in triglyceride from median 138 mg/dl to 115 mg/dl, and an increment in HDLC from median 51 mg/dl to 53 mg/dl, Table 3.

**Table 3:** Follow-up lipid and lipoprotein cholesterol levels after 4 weeks on PCSK9 inhibitor therapy, mean ± SD [median]

	Lipids mg/dl	Pre-treatment	After 4 weeks on PCSK9 inhibitor	Change (mg/dl)	% change	*p* (paired Wilcoxon test)
Of 103 patients, 94 at 4 weeks follow up	TC	251 ± 59 [249]	158 ± 49 [159]	−93 ± 54 [−82]	−36 ± 18 % [−36 %]	<.0001
	TG	163 ± 85 [140]	125 ± 48 [119]	−38 ± 70 [−24]	−16 ± 30 % [−18 %]	<.0001
	HDLC	54 ± 16 [54]	57 ± 17 [55]	+2 ± 9 [+2]	+6 ± 15 % [+4 %]	.0005
	LDLC	166 ± 55 [152]	77 ± 43 [76]	−89 ± 50 [−79]	−52 ± 23 % [−54 %]	<.0001
Of 61 patients with CVD pre study, 56 at 4 weeks follow up	TC	235 ± 56 [230]	148 ± 46 [146]	−88 ± 52 [−80]	−36 ± 19 % [−37 %]	<.0001
	TG	163 ± 97 [138]	118 ± 45 [115]	−44 ± 79 [−31]	−18 ± 29 % [−20 %]	<.0001
	HDLC	54 ± 18 [51]	57 ± 19 [53]	+3 ± 10 [+2]	+7 ± 17 % [+5 %]	.002
	LDLC	151 ± 51 [141]	68 ± 39 [60]	−83 ± 46 [−79]	−54 ± 24 % [−57 %]	<.0001

**Fig. 1:**
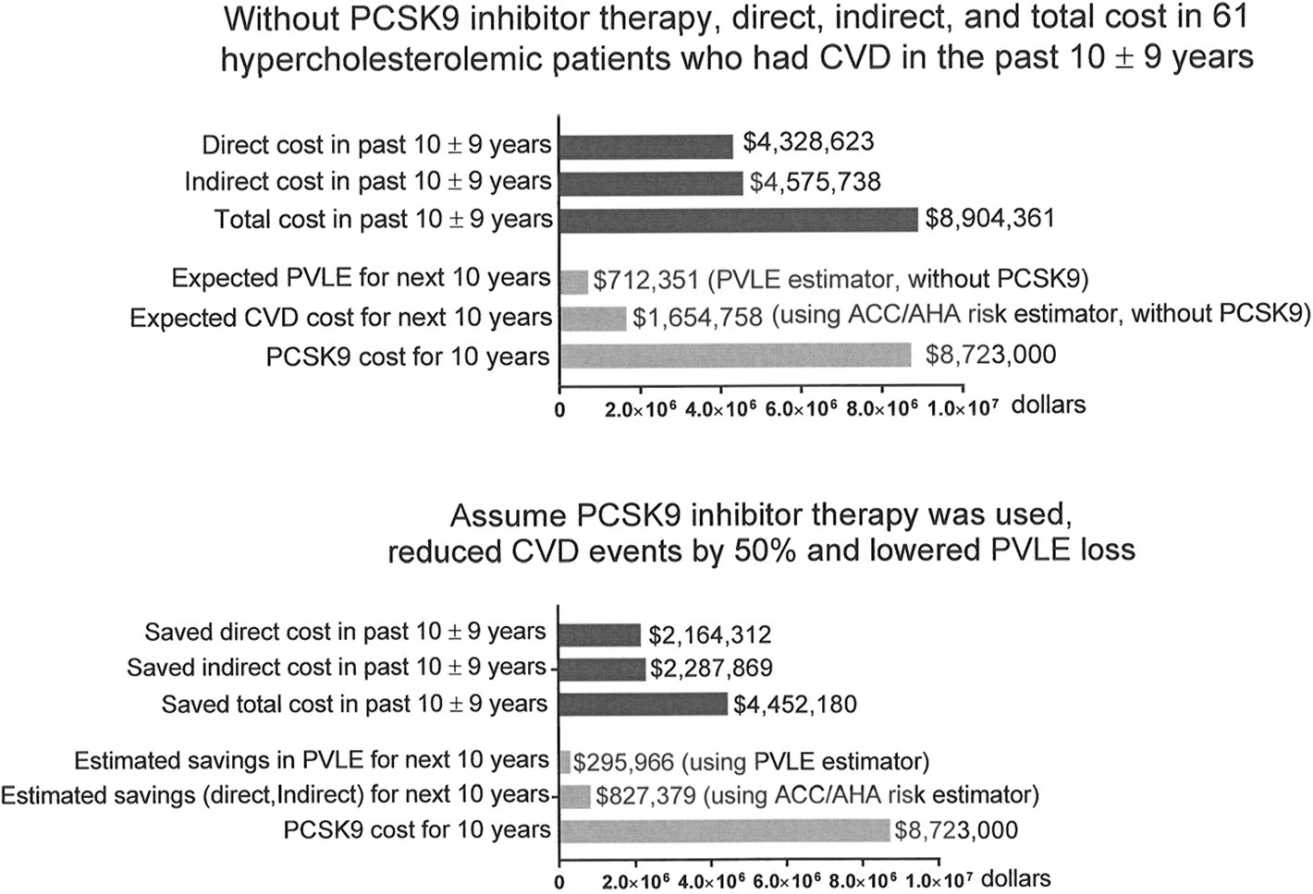
**Top Panel**: Total, direct, and indirect costs, expected CVD costs for the next 10 years, and loss of present value of lifetime earnings (PVLE), and costs of PCSK9 inhibitor therapy in 61 hypercholesterolemic patients who had sustained a cardiovascular event (CVD) in the past 10 ± 9 years. **Bottom Panel**: Assume PCSK9 inhibitor therapy was used and reduced CVD events by 50 % and lowered PVLE loss

Of the 103 patients, 61 had a first CVD event at median age of 55 years and median LDLC 139 mg/dl despite maximal tolerated, non-PCSK9 cholesterol-lowering therapy, Table 1. As displayed in Fig. 1 (top panel), In the 61 patients with CVD events in the past 10 ± 9 years, total direct costs were $4,328,623, with estimated total indirect costs $4,575,738, with total cost $8,904,361. For the 61 patients already having had CVD, future 10-year CVD risk was 15.9 ± 11.7 %, median 13.1 %, calculated using the ACC/AHA calculator (which does not depend on subject’s CVD event history), Table 2. Without PCSK9, expected CVD events in these 61 patients in the next 10 years were estimated to cost $1,654,758, Fig. 1 (top panel), assuming healthcare costs were to stay the same as current.

Using the human capital approach of Menzin et al., [33] we estimated productivity costs as the present value of lifetime earnings (PVLE) lost due to premature mortality, Fig. 1. As displayed in Fig. 1 (top panel), estimated costs of PVLE in the next 10 years in the 61 patients who had already sustained a CVD event was $712,351 without PCSK9 inhibitor therapy. Using PCSK9 inhibitors mortality data by Navarese et al. [34], we estimated savings in PVLE on PCSK9 in the next 10 years of $295,966, Fig. 1 (bottom panel).

Mendelian randomization studies suggest that a lifetime reduction of LDLC ~ 40 mg/dl would reduce risk of ASCVD by 50 % [36]. In our study, after 4 weeks therapy with PCSK9 inhibitors, and beyond maximally tolerated LDLC reduction with diet-statins, median LDLC reduction in the 61 patients with entry CVD was 79 mg/dl, a 57 % reduction (median) from baseline, Table 3. If PCSK9 inhibitors would have reduced ASCVD event rates in the 61 patients with CVD by 50 %, $4,452,180 would have been saved (Fig. 1, bottom panel). If PCSK9 inhibitors were used in the next 10 years, assuming a 50 % reduction in CVD events, savings from the 10 year projected CVD cost would be $827,379, in addition to the estimated savings by reducing lost PVLE $295,966, Fig. 1, bottom panel.

**Fig. 2:**
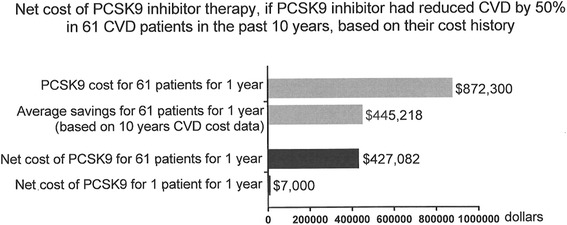
Net cost of PCSK9 inhibitor therapy, based on 61 patients’ cost history, assuming a 50 % reduction in CVD events on PCSK9 inhibitor therapy

In the 61 patients with CVD, PCSK9 therapy costs for 1 year were estimated to be $872,300, Fig. 2. If PCSK9 inhibitor therapy had been used in the past, average savings for these 61 patients due to CVD event rates being halved were estimated to be $445,218 for 1 year (Fig. 2). Net costs for the 61 patients with CVD, were estimated to be $427,082 for 1 year, and net costs per patient per year were estimated to be $7,000 (Fig. 2).

**Fig. 3:**
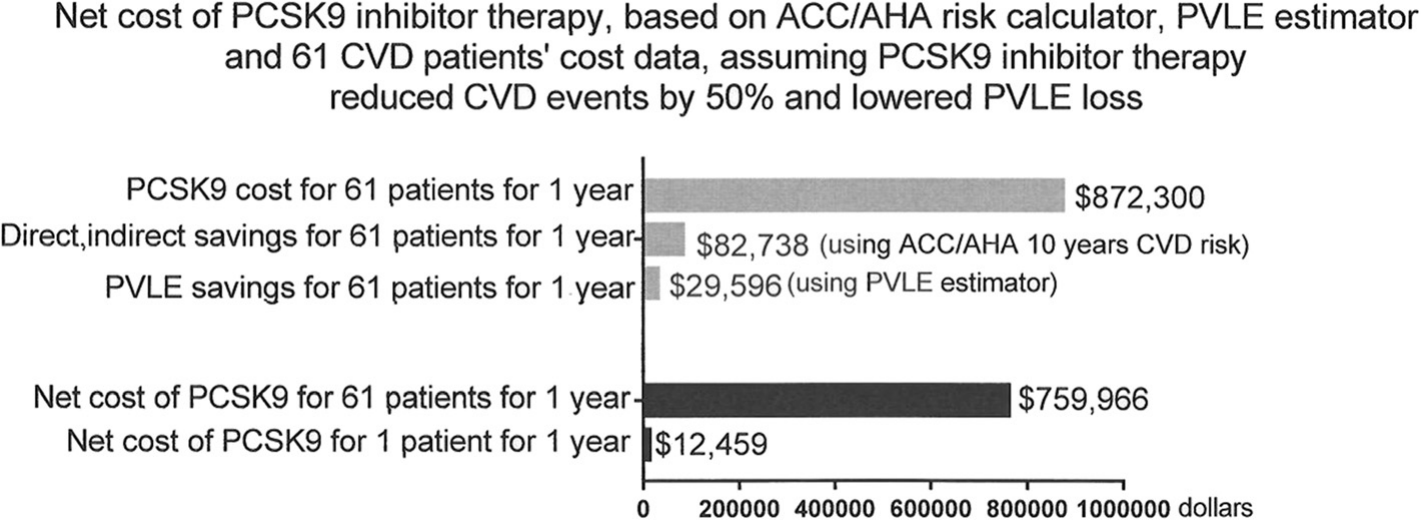
Net cost of PSCK9 inhibitor therapy, based on CVD events estimated from the ACC/AHA risk calculator and present value of lifetime earnings (PVLE) in 61 patients assuming a 50 % reduction in CVD events and lowered PVLE loss on PCSK9 inhibitor therapy

Examining 10 years forward for the 61 patients with CVD before entry, PCSK9 costs for the 61 patients for 1 year were $872,300, Fig. 3. Average annual savings for 61 patients based on halving the estimated 10-year risk of CVD were estimated to be $82,738, and annual savings from otherwise lost PVLE were calculated to be $29,596, Fig. 3. As displayed in Fig. 3, for our 61 patients with previous CVD events, adding estimated savings of reduced CVD events (from the ACC/AHA calculator) and from PVLE, the net cost of PCSK9 therapy was calculated to be $12,459 per patient per year, Table 3.

## Discussion

In the current study, we assessed whether and to what degree PCSK9 inhibitors, as currently priced, would provide an incremental cost-effectiveness ratio (ICER) within a society willingness-to-pay threshold [25, 26], prolonging life, and representing a societal acceptable value. In our 61 patients with CVD at entry, the net cost of PCSK9 inhibitor therapy, assuming a 50 % reduction of CVD events on PCSK9 inhibitor therapy was $7,000 per patient, and the net cost of PCSK9 therapy over the next 10 year period was estimated to be $12,459 per patient per year, well below the $50,000 per year [26] of life saved which has been used to judge value of a pharmacologic therapy. Currently, there is no acceptable model for direct costs of all-cause mortality. Our current ICER shows $7,000 with CVD reduction of 50 %. If it is assumed that on top of CVD reduction of 50 %, all-cause mortality [34] was reduced such that there was 30 % increased savings, then ICER would be $4900 and if it was reduced by 60 % then ICER would be $2800.

In human health-economics there are two major approaches: the human capital approach, and the friction cost method [37–40]. The friction cost method includes all lost productivity costs due to disease mortality only until the deceased worker is replaced by an unemployed worker [41]. The human capital approach is more widely accepted and recommended as it assumes no unemployment, captures all lost productivity due to disease mortality by assuming individuals who died prematurely would have worked full time until the end of their working lives, and includes unpaid labor, such as household work [42–45]. We have used the human capital approach using the model called present value of lifetime earnings (PVLE) to estimate indirect costs due to lost productivity from premature mortality if the patients were not on PCSK9 therapy [33]. Furthermore, the model calculates PVLE based on premature mortality according to age group at death [33]. Presently, direct costs of all-cause mortality cannot be calculated due to lack of acceptable model.

Approximately 735,000 people in US have a myocardial infarction and 795,000 have a stroke each year [20]. Heidenreich et al. project that approximately 41 % of the United States population will have some form of CVD by 2030 [21]. In the MEGA study involving Pravastatin 10 and 20 mg plus diet with follow-up of 5 years, it was demonstrated that CVD risk was reduced by 33-35 %. McConnachie et al. randomized 6,595 hypercholesterolemic patients to pravastatin 40 mg or placebo, and followed them for 15 years [46]. Within five years, in the pravastatin 40 mg group there was saving of NHS £710,000 from CVD related direct costs with gain of 136 quality-adjusted life years [46]. In a rosuvastatin vs. placebo long-term cost effectiveness study with a hypothetical cohort of 100,000 moderate to high risk CVD patients with Framingham risk ≥10 %, there was QALYs (quality-adjusted life-years) gain of 33,480 over life-time, with 9,916 over 10 years [47]. For a quality-adjusted life year gained, ICER was $7062 (lifetime) and $44,466 over 10-years [47]. Montouchet et al. concluded that in a 1,000 member managed care group, statin treatment to goal with rosuvastatin was cost effective, at an additional cost per member per month of only $0.007 [48]. Ademi et al. assessed cost of screening for HeFH and outcomes, assuming a 50 % reduction in events with statins [49]. The ICER ratio was Australian $4155 per years of life saved and $3565 per QALY gained [49]. Aljutaili et al. discussed cost-effectiveness of a CHD preventative program depending on subjects’ risk for CHD, defined as myocardial infarction, stroke or death. They concluded that high risk group would benefit by CHD preventative program (KardioPro) with cost-effectiveness of €20,901- €26,456 per event-free year [50]. Our per year cost-effectiveness $7,000 falls in between numbers suggested by Ademi et al. of AUD 4155 (USD 3,117) on rosuvastatin therapy and Aljutaili et al. of €26,456 (USD 29,101) for high risk/previous CHD event group with all preventative measures.

Statins have been used for 3 decades or more, with the CDC estimating that about 55 % of hypercholesterolemic patients are taking cholesterol lowering therapy, of whom ~90 % are taking statins [19]. Consequently, the American Heart Association estimated that the cost of CVD was $320.1 billion in 2011; they further estimate the cost will rise to $1 trillion by 2030 [22]. Given the rising healthcare cost burden of CVD, which currently excludes potential savings (as in our current report) of PCSK9 inhibitor use, both secondary and primary prevention of CVD [24, 35] will be paramount in the effort to limit the financial burden of CVD on a growing society with limited resources.

Many patients with elevated LDLC cannot achieve treatment targets [1–3] for many reasons [4, 5], while PCSK9 therapy has been shown to be remarkably effective beyond the maximal LDLC lowering achieved by statins [6, 10–13]. In our current study, after maximally tolerated conventional cholesterol lowering therapy, after 4 weeks on PCKS9 inhibitors, median LDLC was decreased to median 79 mg/dl (54 % decrease) from baseline in 103 patients, and was reduced by median 79 mg/dl (57 % decrease) from baseline in the 61 patients with CVD before study entry. Mendelian randomization studies suggest that an approximate 40 mg/dl drop in LDLC over a lifetime reduces risk of ASCVD by 50 % [25, 36]. Maintenance of 79 mg/dl reduction in LDLC observed in the current study should reduce CVD risk by well over 50 %.

With the introduction of powerful PCSK9 inhibitors, and as shown in the current study, many patients can regularly meet LDLC targets, <70 mg/dl for those with CVD [51], reducing future cardiovascular events by an estimated 50 % or more. Roth et al. reported 73 % reduction in LDLC when alirocumab (150 mg given every 2 weeks) was given with atorvastatin (80 mg daily) versus atorvastatin (10 mg daily) alone [52]. Alirocumab ODYSSEY Phase III studies demonstrated that the mean percentage change in calculated LDLC from baseline to week 24 beyond statin effect was −61 % (alirocumab) versus 0.8 % (placebo), *p* < 0.001 [53]. In 2461 patients treated with alirocumab, 796 (32 %) had two consecutive LDLC levels <25 mg/dL while 288 (12 %) had two consecutive LDLC levels <15 mg/dL [13]. Furthermore, In the OSLER-1 and OSLER-2 phase III trials, evolocumab reduced LDL cholesterol levels by -61 % at 12-week on-treatment median [11] beyond statin effect. In a pool of 2651 evolocumab receiving patients, 1609 (61 %) had at-least one LDLC <25 mg/dL. Compared to the placebo, there were minimal adverse reactions to the PCSK9 inhibitors with difference between placebo vs. PCSK9 inhibitor group consistently <2 % [13].

Statin intolerance, predominantly caused by myalgia, myositis, and myopathy occurs in 5 % to 20 % of statin-treated patients [54] who will benefit substantially from PCSK9 inhibitor therapy [55]. In the GAUSS-3 study, 43 % of patients on atorvastatin had muscle symptoms and when these patients were enrolled in Phase B, comparing ezetimibe plus placebo vs evolocumab plus placebo, 29 % had myalgias vs 21 % on evolocumab [55]. Furthermore, LDLC reduction from baseline on ezetimibe was 17 vs. 53 % on evolocumab at 24 weeks. In patients with statin intolerance, evolocumab was well tolerated and effective [55].

PCSK9 inhibitor therapy is reserved as an adjunct to diet and maximally tolerated statin therapy for adults with HeFH or clinical ASCVD requiring additional lowering of LDLC, at a currently listed cost of $14,000–14,600/patient/year. The cost to the society of this drug will rest heavily on the number of people thought to be at high risk with suboptimal cholesterol lowering despite maximally tolerated cholesterol lowering therapy with history of HeFH [56–58] or CVD [59].

Strengths of our study include documented direct costs of CVD events in hypercholesterolemic patients with CVD, as well as calculated indirect costs, both for CVD events in the past, and projected over the forthcoming 10 years. We have used a PVLE model [33] in order to estimate indirect cost of premature mortality if patients were on or not on PCSK9 therapy [34]. This allowed estimations of net costs per year per patient with patients meeting FDA drug-candidate criteria, and receiving PCSK9 inhibitor therapy with either evolocumab or alirocumab. Our direct cost calculations were limited to average hospital costs of having coronary artery disease, acute myocardial infarction, stroke/acute cerebrovascular disease, congestive heart failure, coronary artery bypass graft, percutaneous angioplasty, carotid endartectomy, and follow-up costs for cardiac diagnostic tests (EKG, stress test, Calcium score), office visits, and cardiac rehabilitation. Another limitation of the study involves use of 10 year ACC/AHA risk calculator [35] which does not include weighting factors for patients age >80 years, total cholesterol >320 mg/dL, or previous history of a CVD event. In the current study, we had 61 patients who had previous CVD events, 13 had total cholesterol >320 mg/dL, and 1 was older than 80 year. As a group, we speculate that this subcohort’s 10 year CVD risk was substantially underestimated by the current estimator. Leening et al. suggest incorporation of soft ASCVD outcomes such as ischemic heart failure, transient ischemic attacks (TIAs), angina, and intermittent claudication in calculations of lifetime risks of ASCVD, since a large portion of women and younger individuals suffer from soft ASCVD outcomes [60]. Leening et al. concluded that two-thirds of individuals will develop some form ASCVD over their life span as opposed to one-third dying from ASCVD [60].

## Conclusions

In the 61 CVD patients in our study, net costs would be $7,000 /patient/year if PCSK9 had been used in their past, with a 50 % reduction in CVD event rate. For PCSK9 intervention for the future 10 years, net costs were estimated to be $12,459 /patient/year, both below the $50,000/year quality adjusted life-year a benchmark for value of care [26]. As currently priced, we project that PCSK9 inhibitors will provide an incremental cost-effectiveness ratio within a society willingness-to-pay threshold [25, 26], prolonging life, and representing a societal acceptable value.

## Abbreviations

ACC: American College of Cardiology
AHA: American Heart Association
ASCVD: Atherosclerotic Cardiovascular Disease
BMI: Body Mass Index
CVD: Cardiovascular Disease
FDA: Food and Drug Administration
HDLC: High Density Lipoprotein Cholesterol
HeFH: Heterozgyous Familial Hypercholesterolemia
HoFH: Homozgyous Familial Hypercholesterolemia
LDLC: Low Density Lipoprotein Cholesterol
PVLE: Present Value Life Time Earnings
TC: Total Cholesterol
TG: Triglycerides
